# Dual Tasking for the Differentiation between Depression and Mild Cognitive Impairment

**DOI:** 10.3389/fnagi.2016.00235

**Published:** 2016-10-13

**Authors:** Florian G. Metzger, Markus A. Hobert, Ann-Christine Ehlis, Sandra E. Hasmann, Tim Hahn, Gerhard W. Eschweiler, Daniela Berg, Andreas J. Fallgatter, Walter Maetzler, the TREND Study team

**Affiliations:** ^1^Department of Psychiatry and Psychotherapy, University Hospital TuebingenTuebingen, Germany; ^2^Center for Geriatric Medicine, University Hospital of TuebingenTuebingen, Germany; ^3^Department of Neurodegenerative Diseases, Center of Neurology and Hertie Institute for Clinical Brain Research, University Hospital of TuebingenTuebingen, Germany; ^4^German Center for Neurodegenerative Diseases (DZNE), University Hospital of TuebingenTuebingen, Germany; ^5^Institute of Psychology, Johann Wolfgang Goethe University of FrankfurtFrankfurt, Germany; ^6^Department of Neurology, University Hospital of Schleswig-Holstein Campus KielKiel, Germany

**Keywords:** depression, dual-task costs, gait speed, mild cognitive impairment, working memory

## Abstract

Differentiation of mild cognitive impairment from depression in elderly adults is a clinically relevant issue which is not sufficiently solved. Gait and dual task (DT) parameters may have the potential to complement current diagnostic work-up, as both dementia and depression are associated with changes of gait and DT parameters. Methods: Seven hundred and four participants of the TREND study (Tübinger evaluation of Risk factors for Early detection of NeuroDegeneration) aged 50–80 years were assessed using the Consortium to Establish a Registry for Alzheimer's Disease Plus test battery for testing cognition and Beck's Depression Inventory for evaluation of depression. Based on these results, four groups were defined: acute depressed (*N* = 53), cognitively mildly impaired (*N* = 97), acute depressed, and cognitively mildly impaired (*N* = 15), and controls (*N* = 536). Participants underwent a 20 m walk and checking boxes task under single (ST) and DT conditions. ST and DT performance and dual task costs (DTC) were calculated. Due to the typical age of increasing incidence of depressive and also cognitive symptoms, the 7th decade was calculated separately. Results: ST speeds of gait and checking boxes, DT walking speed, and walking DTC were significantly different between groups. Healthy controls were the fastest in all paradigms and cognitively mildly impaired had higher DTC than depressed individuals. Additionally, we constructed a multivariate predictive model differentiating the groups on a single-subject level. Conclusion: DT parameters are simply and comfortably measureable, and DTC can easily be determined. The combination of these parameters allows a differentiation of depressed and cognitively mildly impaired elderly adults.

## Introduction

During recent years, gait of elderly adults has been extensively studied. Current understanding is that gait is a cognitively controlled task and that, as a result of aging, walking shows decreased automaticity and increased attentional control (Yogev-Seligmann et al., 2008; Boisgontier et al., 2013). This is reflected by reduction of, e.g., gait speed, cadence, stride length, and stride time in elderly compared to young adults (Al-Yahya et al., 2011; Hall et al., 2011). As a consequence, it is not surprising that reduced gait speed is accepted as a clinical parameter and predictor of cognitive impairment and dementia (Ble et al., 2005; Coppin et al., 2006; Holtzer et al., 2006; Verghese et al., 2007).

Based on these observations, it is suggestive that dual tasking (DT) during walking may have the potential to contribute to the diagnosis of clinical and even prodromal phases of dementia. DT is defined as the execution of two actions at the same time; in elderly adults this is often tested with walking while performing a cognitive task, performing two cognitive tasks, or with performing one cognitive and one perception task simultaneously. The most frequently used parameters for the assessment of DT are the speeds of the respective tasks. A particularly powerful parameter is DT costs (DTC), i.e., the relative decrease (in percent) of performance of a task under a DT situation compared to the ST situation. Normal aging as well as mild cognitive impairment (MCI) and Alzheimer's disease (AD) have been associated with impaired DT and increased DTC (Atkinson et al., 2007; Kaschel et al., 2009; Montero-Odasso et al., 2009b; Hall et al., 2011; Muir et al., 2012b). Since “walking while talking” was defined as a predictor of falling (Lundin-Olsson et al., 1997), DT was considered a marker for the interference between cognition and gait.

A first study on the interference of cognition and depression by Kaschel (Kaschel et al., 2009) showed high DTC in AD patients but low ones in depressed and healthy individuals. This result indicates that DTC could indeed be a relevant parameter for the differentiation of cognitive impairment and depression. Both cognitive impairment and depression are very common age-related conditions, and therefore have a high co-incidence (Thomas and O'Brien, 2008; Panza et al., 2011). Older depressed individuals present a MCI associated with a decrease of episodic and semantic memory, processing speed, and most affected, executive function (Alexopoulos, 2003; Baudic et al., 2004). Furthermore, executive dysfunction is a predictor for impairment in DT (Ble et al., 2005; Coppin et al., 2006; Atkinson et al., 2007; Yogev-Seligmann et al., 2008). Executive ability is important for the adaptation in complex environments, e.g., the ability of walking and talking simultaneously or doing any other action necessary in daily life. Executive function seems to be the pivotal point of cognitive function, and especially, of basic DT or even more complex activities of daily living.

Besides the issue of co-incidence of the two conditions, depression is also predictive of cognitive decline: Older adults with a depressive syndrome have a higher risk of future cognitive decline and dementia than non-depressed adults (Byers and Yaffe, 2011).

Therefore, we investigated in this study whether a 20-meter walking task and a simple checking boxes paradigm under ST and DT conditions differentiates older adults with amnestic MCI from those with a depressive syndrome and from those suffering from both of the two conditions. Our hypothesis was that amnestic MCI (aMCI) patients, followed by depressed have higher DTC than healthy older adults with aMCI / depressed patients (aMCI-D) showing highest DTC levels. Moreover, we performed an extra analysis with the population in their 7th decade of life, because this decade includes live events such as retirement and has particularly high incidence rates of depression and cognitive decline and, therefore, an increased need for diagnostic procedures (Palsson and Skoog, 1997).

## Methods

### Subjects

This present study used baseline data from the TREND study (Tübinger evaluation of Risk factors for Early detection of NeuroDegeneration), which is a longitudinal study conducted by the Department of Neurology, Hertie-Institute of Clinical Brain Research and the Department of Psychiatry and Psychotherapy of the University of Tuebingen. Data was collected between March 2009 and May 2010. At the time of assessment, subjects had either none, one or several of the following symptoms, known to occur in the pre-clinical phase of AD: depression, hyposmia, and REM-sleep behavior disorder (RBD)(Berg, 2012). Exclusion criteria were current AD, bipolar affective disorder, schizophrenia, schizoaffective disorder and severe neurological diseases in the medical history such as stroke or epilepsy. Furthermore, intake of Parkinsonian medication, antipsychotic drugs or lithium led to exclusion from the analysis. The TREND study design, sampling, and data collection procedures have been reported elsewhere (Berg, 2012). The study population consisted of 370 female and 334 male individuals aged 50–80 years. Detailed sociodemographic information is provided in Table 1.

**Table 1:** **Demographic and Clinical Parameters of Respective Cohorts**.

**Demographics**	Whole group^*^		**HC**		**C**		**C+D**		**D**		
**Number**	701		536		97		15		53		
**Number 7th decade**	316		240		49		7		20		
	***Mean***	***SD***	***Mean***	***SD***	***Mean***	***SD***	***Mean***	***SD***	***Mean***	***SD***	***p***
Age (years)	63,3	7,2	63,4	7,2	63,8	6,8	59,9	6,3	62,1	7,7	0,136
Education (years)	14,6	2,6	14,7	2,6	14,2	2,7	12,5	1,7	14,2	2,4	0.002
sex (f/m)	369/332		268/268		56/41		9/6		36/17		0.05
**DEPRESSION**											
BDI	7,9	6,7	6,2	4,4	6,6	4,2	21,5	4,6	23,7	5,9	< 0.001
GDS	2,7	3,0	2,0	2,1	1,9	2,1	8,9	3,1	8,5	2,8	< 0.001
ADS-L	11,8	9,3	9,7	7,2	11,7	8,0	26,5	8,1	28,3	9,8	< 0.001
**COGNITION (CERADplus, Z-VALUES)**											
Mini Mental Status Examination (raw data)	28,8	1,2	28,9	1,1	28,2	1,7	28,1	1,9	28,8	1,1	< 0.001
Mini Mental Status Examination	−0,47	1,04	−0,36	0,99	−0,91	1,08	−1,08	1,30	−0,55	1,06	< 0.001
Boston Naming Test	0,28	0,82	0,33	0,79	0,09	0,95	0,01	0,90	0,22	0,79	< 0.001
Word List Immediate Recall	−0,25	1,00	−0,10	0,94	−0,90	0,99	−1,14	1,40	−0,34	0,87	< 0.001
Wordlist Delayed Recall	−0,13	0,93	0,06	0,86	−0,98	0,67	−1,29	0,80	−0,21	0,87	< 0.001
Word List Intrusions	−0,01	0,88	0,03	0,85	−0,14	0,95	−0,26	1,04	−0,04	0,93	0,197
Savings Word List in%	−0,14	1,12	0,05	1,03	−0,98	1,14	−1,07	1,15	−0,29	1,08	< 0.001
Discriminability in%	0,17	0,74	0,25	0,69	−0,13	0,83	−0,19	1,00	0,04	0,78	< 0.001
Figures Immediate Recall	0,18	1,04	0,33	0,86	−0,58	1,45	−0,32	1,31	0,18	1,11	< 0.001
Figures Delayed Recall	−0,01	1,37	0,38	1,05	−2,10	0,87	−1,96	0,89	0,57	1,00	< 0.001
Savings Figures in%	−0,07	1,00	0,17	0,78	−1,44	0,84	−1,28	0,90	0,43	0,89	< 0.001
Verbal Fluency (phonematical)	0,93	1,04	0,98	1,06	0,72	0,95	0,76	0,47	0,85	1,01	0,224
Trail Making Test, part A	0,52	1,13	0,61	1,13	0,18	1,05	−0,29	1,33	0,48	1,08	< 0.001
Trail Making Test, part B	0,33	1,20	0,46	1,18	−0,13	1,16	−0,65	1,13	0,24	1,16	< 0.001
Trail Making Test, part B/part A	−0,17	1,07	−0,13	1,06	−0,29	1,10	−0,45	1,13	−0,23	1,10	0,354

* *Complete data sets, HC, Healthy Controls; C, aMCI; C+D, aMCI and Depressed; D, Depressed*.

Study participants underwent a neurological and neuropsychological examination by trained physicians and study nurses.

According to depressive and cognitive state we defined four groups: healthy controls (HC), aMCI patients (aMCI), aMCI + depression (aMCI-D), and depression (D).

The study was approved by the ethical committee of the Medical Faculty of the University of Tuebingen (Nr. 90/2009BO2). All subjects gave written informed consent in accordance with the Declaration of Helsinki.

### Definition of presence of depression

A score of ≥18 on the Beck's Depression Inventory I was defined as suffering from acute depression (BECK et al., 1961; Arnau et al., 2001: Table 1).

### Cognitive measurements

Trained interviewers administered the CERAD (Consortium to Establish a Registry of Alzheimer's Disease) plus test battery (Morris et al., 1988). This panel contains 7 subtests—among them the Mini Mental Status Examination (MMSE) and the Trail Making Test (TMT)—covering a wide range of cognitive domains, such as memory, language, visuo-spatial abilities, attention, executive function, and global cognitive function. All subtest scores are standardized for gender, age, and education. Results are shown in Table 1.

According to the criteria for amnestic MCI by Petersen, subjects who scored in the mean of the memory domains (delayed recall of the 10-word-list and delayed recall of the four figures) at least one standard deviation below published normative data were classified as (amnestic) MCI (Petersen et al., 1999). Subjects with amnestic MCI have a substantially higher conversion rate to AD compared to undifferentiated MCI (Yaffe et al., 2006), so only subjects with an amnestic MCI according to Petersen were included in the “MCI”-group.

### Gait and DT assessment

Participants walked 20 m twice as fast as possible, once beginning with the right foot, once with the left. The mean of the two values in meter per second was used for further analysis (ST walking speed). ST checking boxes speed was assessed as follows: Participants marked each of 32 boxes on a sheet of paper on a clipboard as fast as possible with a cross using a pen. DT assessment was also performed under “as fast as possible” conditions, without giving any instruction for prioritization to the participant.

Dual-task costs (DTC) are the relative difference of speed of the DT situation compared to the respective ST situation, i.e., the percentage of change of speed from ST to DT. DTC are positive when a loss of speed occurs during the DT situation. According to (Lindemann et al., 2010; Hobert et al., 2011), the following formula was used to calculate DTC of walking and checking boxes, respectively:

DTC=(ST−DT)/ST∗100

### Statistical analysis

#### Univariate analyses

Univariate analyses of covariances (ANCOVAs) were performed for ST, DT and DTC with the between-subject variable of the four groups. The analysis was adjusted for age and gender because these variables were repeatedly shown to have a relevant influence on both walking speed (Jerome et al., 2015) and DT performance (Schaefer, 2014). Results about (influence of) covariates are provided upon request. *Post-hoc* analyses were conducted using *t*-tests for independent samples. No correction for multiple testing was performed due to the exploratory nature of the study. The same analyses as reported above were then conducted for the target group of the 7th decade (60–69 years, *N* = 316). Data analysis was performed with SPSS for windows (17.0.0).

#### Multivariate predictive model

While the univariate analyses outlined above seek to explain variance in the data—thereby enabling hypotheses-testing—this approach does not provide information regarding the potential clinical utility of our findings. To this end, we thus constructed a multivariate predictive model capable of individualized prediction [for a more formal introduction to Predictive Analytics approaches, see (Franklin, 2005)]. Specifically, Gaussian Process Classification (GPC) as described in Marquand et al. (2010) was performed using a customized version of the Gaussian processes for machine learning (GPML) toolbox for Matlab (http://www.gaussianprocess.org/gpml).

To ensure the generalizability of the classifier, we used 20-fold cross-validation to predict a participant's group membership. Sensitivity was indicated by the number of true positives divided by the sum of true positives and false negatives, while specificity was indicated by the number of false negatives divided by the sum of false negatives and true negative cases. Accuracy was computed by summing sensitivity and specificity and dividing by 2.

To establish whether the observed classification accuracy was statistically significant, we ran each classifier 1000 times with randomly permuted labels and counted the number of permutations which achieved higher accuracy than the one observed with the true labels. The *P*-value was then calculated by dividing this number by 1000.

## Results

A significant main effect differencing the four groups HC, aMCI, aMCI-D, and D was found in four experimental parameters, i.e., ST walking speed, ST checking boxes speed, DT walking speed while checking boxes and DTC walking (Table 2).

**Table 2:** **Comparison of single and dual task parameters and dual task costs**.

***Dependent variable***	***df***	***F***	***p***	**η**
Speed fast gait (ST)	3	10.47	< 0.001	0,04
Speed checking boxes (ST)	3	7.92	< 0.001	0,03
Gait speed (DT)	3	8.96	< 0.001	0,04
Speed checking boxes (DT)	3	2.46	0.062	0,01
DTC walking	3	3.42	0.017	0,01
DTC checking	3	1.08	0.355	0,00

*ST, Single Task; DT, Dual Task; DTC, Dual Task Costs*.

**Figure 1:**
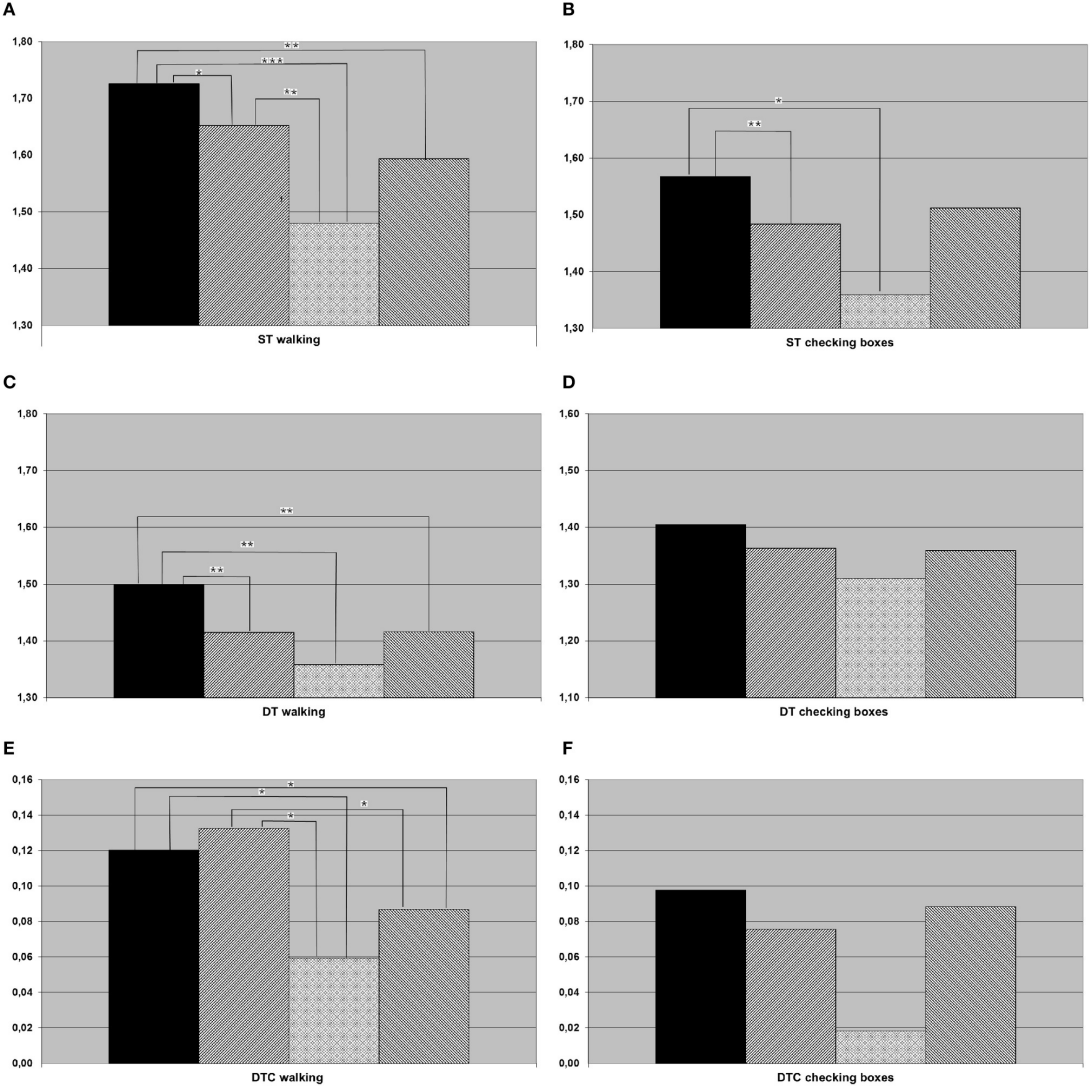
**Results of (A) single task (ST) walking (meters per second), (B) ST checking boxes (crosses per second), (C) dual task (DT) walking, (D) DT checking boxes, (E) dual task costs (DTC) walking, (F) DTC checking boxes for the whole cohort (black column, healthy controls; hatched from bottom left to top right, individuals with amnestic mild cognitive impairment (aMCI); crossed, individuals with aMCI and depression; hatched from bottom right to top left, depressed individuals)**. ^*^p < 0.05, ^**^p < 0.01, ^***^p < 0.001.

In the *post-hoc t*-tests, the following differences between groups were significant: HC were faster compared to all other groups when walking both under ST and DT conditions, and to aMCI and aMCI-D when checking boxes under ST condition. DTC walking were higher in HC than in aMCI-D and D. aMCI patients were faster than aMCI-D in ST walking. DTC walking were higher in aMCI patients than in aMCI-D and D patients. Details are provided in Figure 1.

Sensitivity, specificity, and accuracy for the differentiation of cohorts are presented in Table 3.

**Table 3:** **Sensitivity, Specificity, and Accuracy for the Set of the 6 dual task Parameters**.

	**Sensitivity**	**Specificity**	**Accuracy**
HC vs. C	0.52	0.53	0.53
HC vs. C+D	0.79	0.76	0.77^***^
HC vs. D	0.63	0.56	0.59
C vs. C+D	0.71	0.71	0.71^***^
C vs. D	0.64	0.42	0.53
C+D vs. D	0.66	0.64	0.65^*^

*HC, Healthy Controls; C, aMCI; C+D, aMCI and Depressed; D, Depressed*.

* *p < 0.05*,

*** *p < 0.001*.

**Figure 2:**
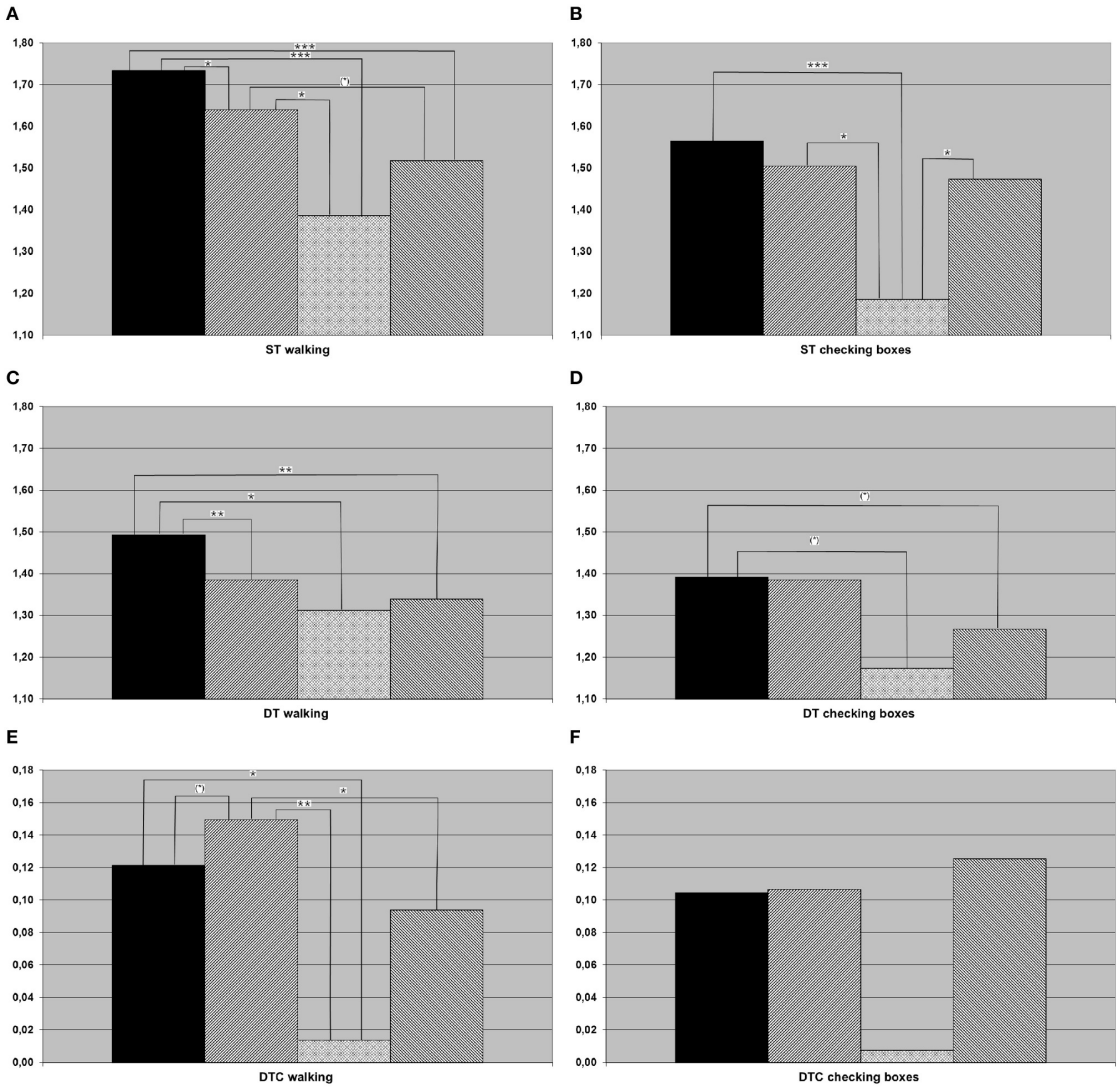
**Results of (A) single task (ST) walking, (B) ST checking boxes, (C) dual task (DT) walking, (D) DT checking boxes, (E) dual task costs (DTC) walking, (F) DTC checking boxes for the population in their seventh decade of life (black column, healthy controls; hatched from bottom left to top right, individuals with amnestic mild cognitive impairment (aMCI); crossed, Individuals with aMCI and depression; hatched from bottom right to top left, depressed individuals**. ^*^p < 0.05, ^**^p < 0.01, ^***^p < 0.001.

Almost all main effects and *post-hoc t*-tests that were significant in the whole cohort were also significant in the 7th decade cohort. The only exception was the main effect differencing the four groups of DTC walking (only significant in the whole cohort). In addition, the following differences were significant in the subcohort but not in the overall cohort: HC checked boxes faster than aMCI-D and D under DT condition, and aMCI had higher DTC walking than HC. aMCI walked faster than D under ST condition, and D checked boxes faster than aMCI-D under ST condition. Details are provided in Figure 2, Table 4.

**Table 4:** **Comparison of single and dual tasking parameters and dual task costs, restricted to the population in the seventh decade of life**.

***dependent variable***	***df***	***F***	***p***	***η*^2^**
Speed fast gait (ST)	3	7.87	< 0.001^***^	0.07
Speed checking boxes (ST)	3	5.63	0.001^**^	0.05
Gait speed (DT)	3	6.76	< 0.001^***^	0.06
Speed checking boxes (DT)	3	2.96	0.032^*^	0.03
DTC walking	3	4.24	0.006^**^	0.04
DTC checking boxes	3	0.79	0.500	0.01

*ST, Single Task; DT, Dual Task; DTC, Dual Task Costs*.

* *p < 0.05*,

** *p < 0.01*,

*** *p < 0.001*.

## Discussion

In this study, speed of walking and checking boxes under ST and DT conditions, as well as DTC when performing both tasks simultaneously were used to test the potential of these parameters to differentiate between the most important psychiatric syndromes of older adults, MCI and depression. A differentiating effect could be shown for the parameters ST walking speed, ST checking boxes speed, DT walking, and DTC walking. The overall effect for the discrimination of aMCI patients from depressed patients was only moderate (see Table 3). However, as the testing is very easy to perform and therefore could simply be implemented in routine clinical testing -only a stop watch, a clipboard with a paper, a pencil and a tape measure are needed- is cheap and does not place unnecessary burden on the participant, we feel that further investigation on its usefulness for the above-mentioned highly relevant clinical differential diagnosis is justified.

The TREND-study cohort is a large sample of older adults with an enriched risk profile. Specifically, more participants than in a population-based cohort have or had depression as a risk factor for neurodegenerative diseases and are currently suffering from a depression. We therefore feel that this cohort is ideal for investigating correlations between cognitive and affective symptoms before manifest dementia. This is relevant as not only cognitive dysfunction but also depression have an impact on gait and DT parameters. Based on literature (Kaschel et al., 2009), it is however possible that gait and DT parameters may be differentially affected by aMCI and depression, suggesting that a quantitative assessment of the former parameters may have the potential to add to this clinically relevant differential diagnosis. Our study indicates that this is indeed true.

Healthy older adults are walking fastest under ST and DT condition, so differentiation between healthy and both aMCI and depressed patients is possible by a simple ST or DT paradigm as it has already and repeatedly been shown by previous studies (Coppin et al., 2006; Holtzer et al., 2006; Verghese et al., 2007; Alexander and Hausdorff, 2008; Montero-Odasso et al., 2009a; Muir et al., 2012a). According to literature, gait speeds under ST and DT conditions are slower in subjects with aMCI than in healthy controls (Montero-Odasso et al., 2009a,b, 2012; Muir et al., 2012b; Jerome et al., 2015). A significant differentiation between MCI and depressed patients was not possible using our ST and DT parameters, indicating that these parameters do not have high potentials to answer our primary study hypothesis. However, DTC of walking could be an interesting marker: This parameter was significantly increased in our aMCI patients, compared to both aMCI-D and depressed patients (Figure 1E). This finding may be best explained by a more cautious behavior of “pure” aMCI patients, compared to the other two groups, under challenging DT conditions. Another interpretation of the result could be that depressed patients (independent of whether they have aMCI or not) are not at their limits when performing ST walking due to e.g., motivational issues, but increase their performance under more challenging conditions. A generally lower gait speed of aMCI-D and D under ST conditions, compared to aMCI as found in this study (see Figure 1A), may support the second hypothesis. Reduced activity and motivation as main symptoms of a depression may partly explain this observation. Motivation seems to play a pivotal role in the development of cognitive decline: Motivation in older adults has been discussed as protective factors for cognitive and depressive symptoms for at least one decade, but without discussing a different impact of motivational aspects on cognitive decline or depression (Forstmeier and Maercker, 2008; Baer et al., 2013). In contrast to this discussion and our results, Kaschel et al. described no DT impairment in depressed participants (while finding an effect in Alzheimer's patients) in two different DT experiments (Kaschel et al., 2009). Age as a possibly strong factor was similar in the former and this study, thus this aspect cannot explain the differences observed in respective studies. An important aspect that is different to our study is the definition of depression as a chronic disease excluding e.g., late onset depression with white matter lesions, which also can influence motivation. The group of depressed subjects in the present study is characterized by acute symptoms of depression as defined by a self-rating questionnaire. Furthermore, the DT paradigm of Kaschel et al. consists of two cognitive tasks, and walking is no element of investigation. Gait on its own is a complex motion task which differs from usual cognitive tasks and, therefore, might produce different results in DT paradigms including gait tasks. In a study based on a DT paradigm with walking and a trail making test, subjects with a major depression showed significantly higher DTC than controls (Wright et al., 2011). However, in this study, DTC was defined as the relation between a simple and an advanced DT paradigm, whereas the conventional DTC uses ST and DT speed.

Another cause for the decreased ST and DT gait speed in depressed subjects in the present study may be medication in terms of a decelerating factor which was not considered in this study due to the heterogeneity of the antidepressants. In a large population based study, an influence of antidepressants (but not of depression itself) on DT parameters was found (Schaefer, 2014). Furthermore, Parkinsonian medication or antipsychotics may have potentially an even larger influence on ST, DT, and DTC; however, the use of these drugs was an exclusion criterion and therefore cannot account for any observation made in the study.

Executive function is considered an important link between depression and cognition. Two studies independently describe the direct relation between executive function, gait speed and DTC (Coppin et al., 2006; Yogev-Seligmann et al., 2008). Furthermore, executive function is impaired in patients with depression (Veiel, 1997). Reduced gait speed as a marker for decreased executive function was also found in depressed subjects in this study, basically confirming the above-mentioned results. Surprisingly, DTC in depressed subjects were not higher—as expected—but lower compared to healthy controls. Our observation may be partly explained by motivational aspects or distraction from rumination or fear but should be specifically investigated in future studies, to re-evaluate our current understanding of the interaction of executive function, depression and gait.

The multivariate modeling approach enabling individualized prediction indicates a potential clinical utility of our measures, particularly concerning the individuals of the group C+D in contrast to all other groups. Moreover, improving single-subjects prediction level needs probably an implementation of more variables than the investigated set.

The exploratory analysis of the cohort in the 7th decade, which is—from a clinical perspective—the most important decade for differential diagnosis between depression and cognitive impairment associated with neurodegenerative diseases (Palsson and Skoog, 1997), revealed an additional aspect: ST speed of checking boxes was significantly lower in the aMCI-D cohort than in both, aMCI and depressed patients. The performance of a fine motor test with visual input may therefore be a potentially interesting test to differentiate this cohort from other disease states. This is, to the best of our knowledge, the first parameter described in the literature for this specific aspect. In conjunction with the strikingly (but not significantly) low DTC of checking boxes (Figure 2F), this result also argues for the hypothesis that depressed patients with aMCI may exhibit an altered motivational / attentional status depending on the complexity of instructions given.

A limitation of this study is that current state of depression is based on the score of a self-rating questionnaire. No external rating of the depressive symptoms was performed; therefore variations in daily state possibly influence the results. Another limitation concerns the heterogeneous group sizes with a large range and the small number of participants suffering from both, MCI and depression (*N* = 15; *N* = 536 in the group of healthy controls, *N* = 97 in the MCI group and *N* = 53 in the depressive group). Therefore, results concerning particularly the latter group should be treated with caution, especially in the 7th decade.

In conclusion, the present study is, to our best knowledge, the first investigating the potential of a challenging DT paradigm to differentiate aMCI and current depression, a relevant clinical differential diagnosis in older adults. Results are encouraging and indicate that the intra-individual comparison between ST and DT performance can delineate performance strategies that differentiate patients with aMCI and depression. Longitudinal studies are needed to confirm results of this exploratory analysis.

## Authors contributions

Study design: WM, AF, DB, GE; Data acquisition: SH, MH, WM; analysis and interpretation: FM, AE, TH, MH, WM. Manuscript drafting: FM, MH, AE, SH, TH, GE, DB, AF, WM. Final approval: FM, MH, AE, SH, TH, GE, DB, AF, WM. Agreement to be accountable: FM, MH, AE, SH, TH, GE, DB, AF, WM

## Funding

AE was partly supported by the IZKF Tübingen (Junior Research Group 2115-0-0). WM reports grants from EU project Moving beyond, SENSE-PARK; Neuroalliance, MJFF and personal fees from Licher, Roelke Pharma, UCB, and GSK. DB is member of an Advisory Board of UCB pharma GmbH and receives honoraria from UCB pharma GmbH. She reports grants from Michael J. Fox Foundation, Janssen Pharmaceutica N.V., German Parkinson's Disease Association (dPV), BMWi, BMBF, Parkinson Fonds Deutschland gGmbH, UCB Pharma GmbH, TEVA Pharma GmbH, EU, Novartis Pharma GmbH, Boehringer Ingelheim Pharma GmbH, Lundbeck. MH received travel grants by Abbvie and Merz. All other authors report no conflict of interest or funding.

### Conflict of interest statement

The authors declare that the research was conducted in the absence of any commercial or financial relationships that could be construed as a potential conflict of interest.

## Abbreviations

aMCI: Amnestic mild cognitive impairment
D: Depression
DT: Dual task
DTC: Dual task costs
MCI: Mild cognitive impairment
ST: Single task
